# Biatrial performance in children with hypertrophic cardiomyopathy: CMR study

**DOI:** 10.1007/s00330-018-5519-7

**Published:** 2018-06-07

**Authors:** Łukasz Mazurkiewicz, Lidia Ziółkowska, Joanna Petryka, Mateusz Śpiewak, Łukasz Małek, Agata Kubik, Magdalena Marczak, Jolanta Misko, Grażyna Brzezińska-Rajszys

**Affiliations:** 1 Departament of Cardiomyopathies, CMR Unit, Institute of Cardiology, 42nd Alpejska Str, 04-682 Warsaw, Poland; 2 Department of Pediatric Cardiology, The Children’s Memorial Health Institute, Warsaw, Poland; 3 Department of Coronary and Structural Heart Diseases, CMR Unit, Institute of Cardiology, Warsaw, Poland; 4 CMR Unit, Institute of Cardiology, Warsaw, Poland; 5 Institute of Cardiology, Warsaw, Poland

**Keywords:** Hypertrophic cardiomyopathy, Heart atria, Fibrosis, Strain, Children

## Abstract

**Objectives:**

To investigate biatrial mechanics and their relation with left ventricular outflow tract (LVOT) obstruction (LVOTO), the degree of hypertrophy, indices of ventricular diastolic function and fibrosis in children with hypertrophic cardiomyopathy (HCM).

**Methods:**

Fifty-five consecutive, prospectively recruited children with HCM (mean age 12.5 ± 4.6 years, 69.1% male), 19 (34.5%) of whom had LVOTO, underwent cardiac magnetic resonance and echocardiography with quantification of phasic components of biatrial function, biventricular diastolic function and fibrosis. Twenty healthy, sex-matched subjects served as controls.

**Results:**

We found a significant increase of left atrial (LA) and right atrial (RA) volumes and reduction in the majority of indices of contractile function, strains and strain rates (*p* < 0.05) in children with HCM compared with controls. Nearly all of the LA dynamics markers attained a significant association with the LVOT gradient (*p* < 0.05), the RA volumes and contractile functions were affected by LV fibrosis and mass (*p* < 0.05), and the RA mechanical components were related to the degree of LVOTO (*p* < 0.05). The minority of biatrial dynamics markers were associated with indices of ventricular diastolic function.

**Conclusions:**

The majority of biatrial volumetric and functional indices were severely compromised in children with HCM compared with controls. The degree of LVOTO appears to trigger LA volumetric and LA and RA mechanical malfunction. On the other hand, the deterioration of RA volumetric components was linked to LV fibrosis and mass.

**Key Points:**

*• Biatrial function was severely compromised in children with HCM.*

*• Left atrial malfunction was associated with the degree of LVOTO.*

*• Fibrosis and LV mass were related to RA volumetric and contractile dysfunction.*

*• The degree of LVOTO was linked to right atrial mechanical abnormalities.*

## Introduction

Hypertrophic cardiomyopathy (HCM) is a primary, genetically triggered myocardial disease characterised by uncontrolled left ventricular (LV) muscle proliferation, diverse clinical presentation and significant outcomes [1, 2]. Its complex pathomorphology includes fibre disarray, microvasculature abnormalities and vast fibrosis [3]. All these serve to decrease LV compliance and result in abnormal LV relaxation and a restrictive filling pattern [4]. The consequent elevation in LV end-diastolic pressure is transmitted back to the left atrium (LA) and further through pulmonary circulation into the right ventricle (RV) and right atrium (RA), which result in dilatation and decreased atrial performance. Left atrium size has been found to be a crucial index of cardiac malfunction used to grade HCM severity and stratify the risk of sudden cardiac death [5–8].

Cardiovascular magnetic resonance (CMR), in both adults and children, has emerged as an adjunctive imaging modality that may, besides yielding volumetric analysis, provide deeper insights into the distribution and degree of cardiac hypertrophy and fibrosis [9–11]. Moreover, CMR-derived strains as markers of cardiac displacement may deliver additional data of myocardial performance. Nowadays, owing to modern feature-tracking (FT) technology, CMR is devoid of the disadvantages of classical tagging technique, which was not suitable for evaluating thin myocardial structures such as atrial walls [12, 13]. Kowallick et al. demonstrated the feasibility of applying CMR FT for analysing biatrial mechanics in healthy subjects [14]. They also reported that fibrosis played a larger role than hypertrophy in the development of LA dysfunction in adults with HCM [15]. Despite this progress, contemporary knowledge of LA and especially RA dynamics in a juvenile population with HCM remains rudimentary. The progressive nature of hypertrophy and fibrosis limits the application of Kowallick et al.’s conclusions directly from adults to younger populations. Therefore, this study focuses on investigating biatrial performance and its relation to LV outlet tract obstruction (LVOTO), biventricular diastolic function and fibrosis in children with HCM.

## Materials and methods

This study was approved by the institutional ethics committee and written informed consent to participate in the study was obtained from all of the subjects and their parents.

The study cohort comprised 55 consecutive, prospectively recruited children with HCM, 19 of whom had LVOTO. Criteria for inclusion in the study included an age younger than 18 years at the time of diagnosis and echocardiographic evidence of LV hypertrophy defined as a diastolic septal thickness or LV diastolic wall thickness z-score greater than 2 [determined as more than two standard deviations from the mean value for the population corrected for body surface area (BSA)] in the absence of haemodynamic conditions that could account for the observed hypertrophy [2].

Normal values of CMR volumetric and mechanical parameters were sourced from the scans of 20 healthy sex-matched, young adult volunteers with no significant medical history, normal physical examination and normal 12-lead ECG.

### CMR examination

A standard CMR study was performed using a 1.5-Tesla scanner (Sonata and Avanto, Siemens, Erlangen, Germany). Late gadolinium enhancement (LGE) images in long-axis and short-axis imaging planes were obtained with a breath-hold, segmented inversion recovery sequence performed 10 min after contrast injection (gadobutrol, Gadovist, Bayer, Germany and gadoteridol, Prohance, Takeda, Japan). The control subjects did not receive gadolinium contrast agent.

### Echocardiographic examination

Two-dimensional, conventional pulsed Doppler and M-mode echocardiography was performed at rest using standard methods (iE 33, Philips, Healthcare). Conventional pulsed Doppler was used to record the mitral and tricuspid inflow patterns at the leaflet tips in the apical four-chamber view. Peak velocities of E and A waves (cm/s) and their ratio (E/A) were measured.

To determine maximal degree of LVOTO, two-dimensional and Doppler echocardiography was performed during the Valsalva manoeuvre in the sitting position, and then during standing if no gradient was provoked. A maximum gradient greater than 30 mmHg was considered significant. Neither pharmacological stimuli nor exercise tests were used to determine maximal LVOT gradients. Furthermore, CMR cine images were visually assessed for the presence of LVOTO.

### Image analysis

Steady-state free precession images were used for calculating ventricular volumes and ejection fractions with the aid of dedicated software (MASS 7.6, Medis, Leiden, Netherlands). Manual delineation of endocardial and epicardial contours was performed in end-diastolic and end-systolic phases. Left ventricular end-diastolic volume (LVEDV), LV end-systolic volume (LVESV), LV mass (LVM) and LV ejection fraction (LVEF) were calculated. LVEDV, LVESV and LVM were indexed to BSA (LVEDVI, LVESVI and LVMI, respectively). Also, on the basis of two- and four-chamber views, maximal LV wall thickness was measured. Images were visually assessed for the presence of LGE areas for each LV myocardial segment using the 17-segment cardiac model. In addition, the quantitative extent of LV LGE was determined using dedicated software (MASS 7.6, Medis). Regions of elevated signal intensity had to be confirmed in two spatial orientations. A region of interest (ROI) was selected in effectively nulled myocardium. The mean signal intensity and SD of the ROI were measured. The LV myocardium was delineated by endocardial and epicardial contours, which were traced manually. Enhanced myocardium was defined as myocardium with a signal intensity exceeding 6 SDs above the mean of the ROI. The extent of LGE was expressed as a percentage of the LV mass (LGE%LV).

Left atrial and RA volumes were quantified using dedicated software (MASS 7.6, Medis). The LA volumes were calculated according to the biplane area–length method [16]. Manual tracking of the LA area and length was performed in two- and four-chamber views excluding pulmonary veins and the LA appendage. The RA volumes were calculated according to the single-plane area–length method [17]. Manual tracking of RA area and length was performed in four-chamber view. The LA volumes, indexed for BSA, were assessed at LV end-systole (LAV max), at LV diastole before LA contraction (LAV pac) and at late LV diastole after LA contraction (LAV min). The RA volumes, indexed for BSA, were assessed at RV end-systole (RAV max), at RV diastole before RA contraction (RAV pac) and at late RV diastole after RA contraction (RAV min). The LA and RA volumetric analysis was performed twice by two independent and skilled observers. Total atrial emptying fraction (LAEF total, RAEF total corresponding to LA and RA reservoir, respectively), passive atrial emptying fraction (LAEF passive, RAEF passive corresponding to LA and RA conduit function, respectively) and active atrial emptying fraction (LAEF booster, RAEF booster corresponding to LA and RA contractile booster pump function, respectively) were defined according to the following equations:

$$ \mathrm{EFtotal}=\left(\mathrm{Vmax}\hbox{--} \mathrm{Vmin}\right)\times 100/\mathrm{Vmax} $$

$$ \mathrm{EFpassive}=\left(\mathrm{Vmax}\hbox{--} \mathrm{Vpac}\right)\times 100/\mathrm{Vmax} $$

$$ \mathrm{EFbooster}=\left(\mathrm{Vpac}\hbox{--} \mathrm{Vmin}\right)\times 100/\mathrm{Vpac} $$

### Feature tracking analysis

**Fig. 1:**
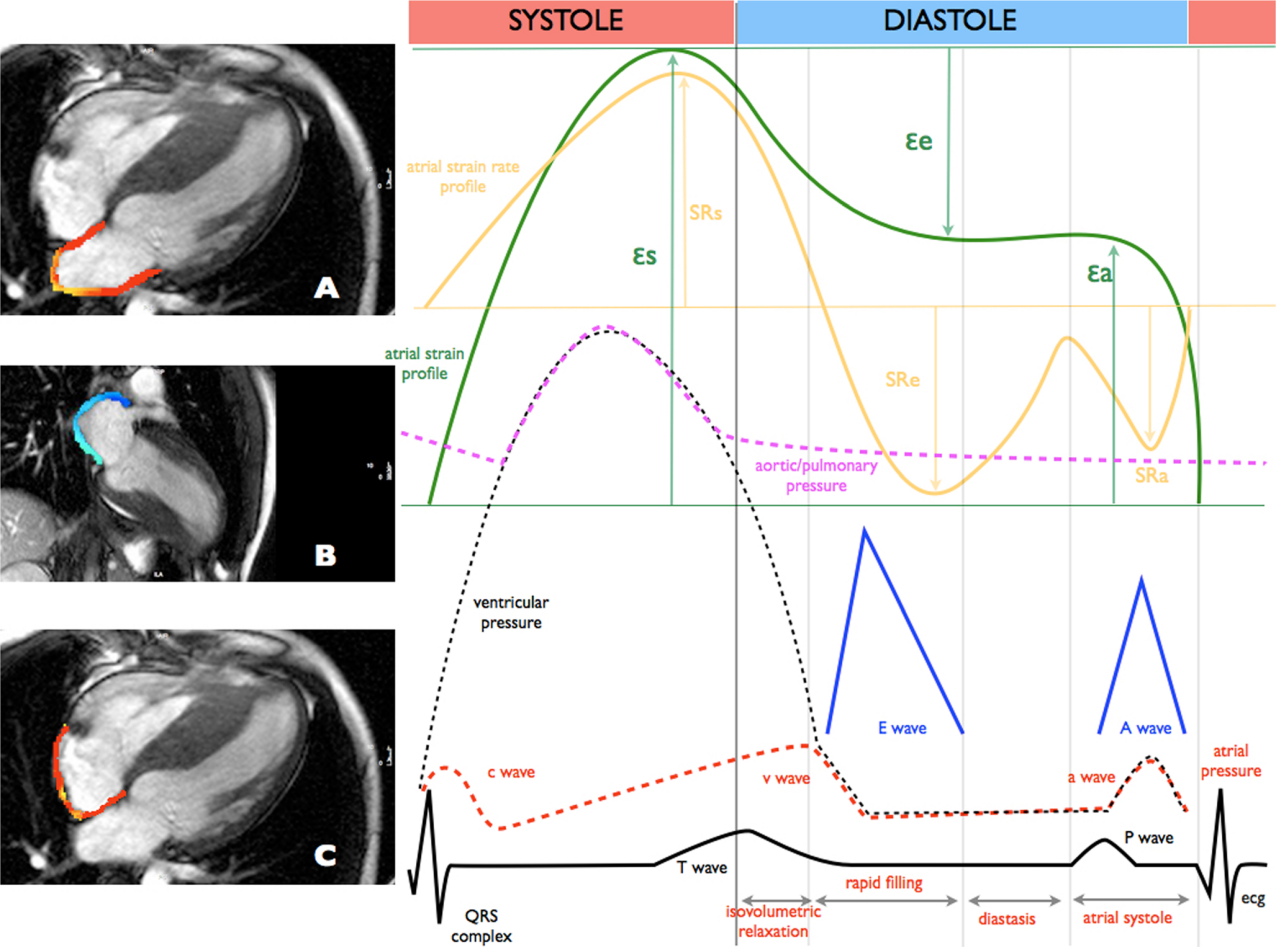
Left atrial myocardial tissue tracked in two- and four-chambers view (**a**, **b**) and right atrial myocardial tissue tracked in four-chamber view (**c**) for CMR feature tracking analysis. Example of functional curves for quantification of atrial strains and strain rates superimposed onto the ECG, Doppler inflow characteristics, atrial and ventricular pressure waveforms. Reservoir aspect includes total strain (εs) and peak positive strain rate (SRs). Conduit aspect includes passive strain (εe) and peak early negative strain rate (SRe). Contractile booster pump function includes active strain (εa) and peak late negative strain rate (SRa). Atrial strain waveform is marked green and strain rate yellow. Solid lines are ECG (black), transmitral/tricuspid inflow velocities (blue). Dashed lines are pressure curves: atrial (red), ventricular (black), aortic/pulmonary (purple)

Atrial strains and strain rates were analysed using dedicated software (Cvi42, Alberta, Canada). Left atrial endocardial borders were tracked in two- and four-chamber views. Right atrial borders were tracked in four-chamber view (Fig. 1). The atrial endocardial border was manually delineated in diastolic phase and tracked automatically. Three aspects of LA and RA mechanics were analysed: passive strain (εe, corresponding to atrial conduit function), active strain (εa, corresponding to atrial contractile booster pump function) and total strain, the sum of the passive and active strains (εs, corresponding to atrial reservoir function). Accordingly, three strain rate parameters were evaluated: the peak positive strain rate (SRs, corresponding to atrial reservoir function), the peak early negative strain rate (SRe, corresponding to atrial conduit function) and the peak late negative strain rate (SRa, corresponding to atrial contractile booster pump function).

### Statistical analysis

All of the continuous variables were expressed as mean ± SD or as median and interquartile range and were tested for normal distribution using the Kolmogorov–Smirnov test. Comparisons between groups were performed using the Student’s *t* test or the Wilcoxon–Mann–Whitney *U* test for continuous variables and the chi-square or Fisher’s exact test for categorical variables, as appropriate.

Intraobserver and interobserver variability of the indices of atrial performance and quantitative analysis of LV fibrosis were evaluated using the Bland*–*Altman test. The reproducibility analysis was performed by two experienced operators (LMaz, JP). Duplicability analysis was assessed using intraclass correlation coefficients (ICCs) and coefficients of variation (CoVs).

Linear, multivariable regression analysis was conducted separately in three models, each for LA and RA, to determine the association between each index of atrial performance and the LVOT gradient (model adjusted for age, sex, LVMI, LGE%LV, maximal wall thickness and β-blocker use), indices of atrial performance vs LGE%LV (model adjusted for age, sex, LVMI, LVOT gradient, maximal wall thickness and β-blocker use) and indices of atrial performance vs LVMI (model adjusted for age, sex, LVOT gradient, LGE%LV, maximal wall thickness and β-blocker use). Correlations between continuous variables were tested using Spearman correlation coefficients. The variables with *r* > 0.50 were not included in the same multivariable model.

A two-sided *p* value less than 0.05 was considered to indicate statistical significance. The statistical analyses were performed using MedCalc 12.1.4.0 software (MedCalc, Mariakerke, Belgium).

## Results

As expected, children with HCM had higher myocardial mass indexed to BSA (*p* = 0.01) and greater maximal LV wall thickness (*p* < 0.01) compared with controls. By qualitative evaluation, fibrosis was detected in 27 patients (41.1%). Of those 12 children (21.8%) had LGE in RV insertion points, while in 15 subjects (27.1%) diffuse fibrosis was observed. The diffuse collagen deposits were mainly localised in hypertrophied parts of myocardium. Visually, there was no scarring within the RV myocardium. The fibrosis extent was 1.76 ± 3.2% of the LV mass. Table 1 compares the baseline characteristics of the HCM subjects and the controls. Juveniles with obstructive HCM exhibited larger maximal LV wall thicknesses (*p* < 0.01), larger LA size (*p* < 0.01) and more fibrosis (*p* = 0.02) than individuals without LVOTO. The baseline demographic and CMR data for subjects with and without LVOTO are presented in Table 2.

**Table 1:** Baseline demographic, clinical and volumetric data in HCM subjects and controls

	Study group *n =* 55	Controls *n =* 20	*p*
Age (years)	12.5 ± 4.6	24.8 ± 5.2	0.03
Male sex, *n* (%)	38 (69.1)	13 (66.6)	NS
BSA (m^2^)	1.5 ± 0.2	1.8 ± 0.2	NS
NYHA	1.7 ± 0.5	NA	NA
MRI parameters			
LVEDVI (ml/m^2^)	79.7 ± 17.5	85.9 ± 10.2	< 0.01
LVESVI (ml/m^2^)	27.2 ± 10.1	31.1 ± 5.3	< 0.01
LVSVI (ml/m^2^)	52.5 ± 11.5	58.8 ± 7.4	< 0.01
LVEF (%)	65.8 ± 7.4	68.4 ± 6.2	NS
LVMI (g/m^2^)	94.9 ± 59.7	73.1 ± 19.3	0.01
Maximal wall thickness (mm)	19.1 ± 8.4	8.7 ± 2.1	< 0.01
LAA (cm^2^)	19.9 ± 9.2	15.3 ± 3.5	< 0.01
Areas of LGE (%)	27 (49.1)	NA	NA
LGE%LV	1.76 ± 1.8	NA	NA
MR (ml)	9.4 ± 4.1	1.3 ± 3.1	< 0.01
RVEDVI (ml/m^2^)	73.4 ± 13.5	80.1 ± 15.4	0.02
RVESVI (ml/m^2^)	27.7 ± 11.2	26.4 ± 11.2	NS
RVSVI (ml/m^2^)	45.7 ± 12.2	53.7 ± 13.8	0.01
RVEF (%)	62.2 ± 8.7	67.1 ± 9.7	NS
RVMI (g/m^2^)	24.4 ± 4.7	20.3 ± 4.1	NS
Biventricular diastolic function indices			
Transmitral E wave velocity (cm/s)	87.4 ± 28.6	NA	
Transmitral A wave velocity (cm/s)	56.1 ± 16.5	NA	
Transmitral E/A	1.7 ± 0.9	NA	
Transtricuspid E wave velocity (cm/s)	60.1 ± 14.6	NA	
Transtricuspid A wave velocity (cm/s)	41.3 ± 10.5	NA	
Transtricuspid E/A	1.5 ± 0.5	NA	

*BSA* body surface area, *LVEDVI* left ventricle end-diastolic volume index, *LVESVI* left ventricle end-systolic volume index, *LVSVI* left ventricle stroke volume index, *LVEF* left ventricle ejection fraction, *MR* mitral regurgitation, *RVEDVI* right ventricle end-diastolic volume index, *RVESVI* right ventricle end-systolic volume index, *RVSVI* right ventricle stroke volume index, *RVEF* right ventricle ejection fraction, *FT* feature tracking, *LGE* late gadolinium enhancement, *LGE%LV* amount of fibrosis as a percentage of LV mass, *NA* not applicable, *NS* not significant

**Table 2:** Baseline demographic and CMR data in subjects with obstructive and non-obstructive forms of hypertrophic cardiomyopathy

	Non-obstructive HCM *n =* 34	Obstructive HCM *n =* 19	*p*
Age (years)	12.1 ± 3.8	13.2 ± 4.7	NS
Male sex, *n* (%)	23 (69.1)	13 (68.4)	NS
BSA (m^2^)	1.3 ± 0.2	1.5 ± 0.2	NS
NYHA	1.3 ± 0.5	1.9 ± 0.4	0.01
LVOT gradient (mmHg)	10.6 ± 6.9	75.8 ± 23.5	< 0.01
MRI parameters			
LVEDVI (ml/m^2^)	77.4 ± 17.9	83.9 ± 15.2	NS
LVESVI (ml/m^2^)	27.7 ± 6.9	26.9 ± 11.3	NS
LVSVI (ml/m^2^)	49.7 ± 10.5	57 ± 12.4	NS
LVEF (%)	64.2 ± 7.4	69.8 ± 6.2	NS
LVMI (g/m^2^)	89.2 ± 66.1	112.1 ± 39.4	NS
Maximal wall thickness (mm)	16.4 ± 6.7	23.1 ± 8.1	< 0.01
LAA (cm^2^)	15.7 ± 6.1	25.7 ± 9.7	< 0.01
Areas of LGE (%)	11 (32.3)	16 (84.2)	< 0.01
LGE%LV	1.4 ± 1.5	2.5 ± 2.2	0.02
MR (ml)	7.5 ± 3.6	13.2 ± 4.1	< 0.01
RVEDVI (ml/m^2^)	62.9 ± 13.4	84.1 ± 15.7	< 0.01
RVESVI (ml/m^2^)	24.1 ± 8.6	31.4 ± 11.5	NS
RVSVI (ml/m^2^)	38.8 ± 11.4	52.7 ± 10.3	0.02
RVEF (%)	61.6 ± 8.9	62.6 ± 10.3	NS
RVMI (g/m^2^)	23.9 ± 4.7	24.9 ± 5.1	NS

*BSA* body surface area, *LVEDVI* left ventricle end-diastolic volume index, *LVESVI* left ventricle end-systolic volume index, *LVSVI* left ventricle stroke volume index, *LVEF* left ventricle ejection fraction, *LVOT* left ventricular outflow tract, *MR* mitral regurgitation, *RVEDVI* right ventricle end-diastolic volume index, *RVESVI* right ventricle end-systolic volume index, *RVSVI* right ventricle stroke volume index, *RVEF* right ventricle ejection fraction, *LAA* left atrial area, *LGE%LV* amount of fibrosis as a percentage of LV mass, *RVMI* right ventricular mass indexed for body surface area, *NS* not significant

### Biatrial performance in HCM children and controls

The comparisons of volumetric and mechanical components of biatrial function between children with HCM and healthy controls are presented in Table 3. All LA volumes were higher in HCM subjects than in the controls (*p* = 0.01–0.02). Furthermore, children with HCM had conduit (*p* = 0.04) and booster (*p* = 0.03) fractional volume changes significantly lower than those of the controls. There were no differences in strain or strain rate booster functions between HCM juveniles and controls (*p* = not significant, NS); all other LA mechanics indices were significantly compromised in the study group compared with the controls (*p* = 0.01–0.04).

**Table 3:** Comparisons of biatrial volumetric, contractile and mechanical components between children with HCM and controls

	Controls *n =* 20	Study group *n =* 55	*p*		Controls *n =* 20	Study group *n =* 55	*p*
LAV min (ml/m^2^)	15.3 ± 12.4	22.4 ± 9.7	0.02	RAV min (ml/m^2^)	11.7 ± 4.6	17.7 ± 8.3	< 0.01
LAV max (ml/m^2^)	38.2 ± 10.5	44.7 ± 8.6	0.02	RAV max (ml/m^2^)	30.3 ± 5.1	32.1 ± 13.3	NS
LAV pac (ml/m^2^)	26.2 ± 9.5	34.6 ± 11.6	0.01	RAV pac (ml/m^2^)	21.1 ± 5.1	27.6 ± 11.3	0.01
LAEF reservoir (%)	59.7 ± 19.2	54.2 ± 12.6	NS	RAEF total (%)	61.8 ± 8.2	47.1 ± 12.1	< 0.01
LAEF conduit (%)	32.6 ± 12.1	25.7 ± 11.7	0.04	RAEF passive (%)	30.2 ± 11.2	18.2 ± 12.6	< 0.01
LAEF booster (%)	46.6 ± 9.4	37.1 ± 11.1	0.03	RAEF booster (%)	43.7 ± 8.5	37.5 ± 12.3	0.04
LA εS (%)	23.6 ± 5.9	18.6 ± 6.3	0.01	RA εS (%)	23.6 ± 4.6	18.7 ± 6.1	0.01
LA εE (%)	16.5 ± 5.9	11.9 ± 5.3	0.01	RA εE (%)	17.9 ± 3.7	11.6 ± 5.4	< 0.01
LA εA (%)	7.1 ± 3.7	6.6 ± 4.6	NS	RA εA (%)	5.7 ± 2.1	7.1 ± 3.5	0.04
LA SRs (1/s)	1.2 ± 0.3	1.0 ± 0.3	0.04	RA SRs (1/s)	1.2 ± 0.3	1.0 ± 0.4	NS
LA SRe (1/s)	– 1.3 ± 0.4	– 1.0 ± 0.3	0.03	RA SRe (1/s)	– 1.7 ± 0.6	– 0.9 ± 0.4	< 0.01
LA SRa (1/s)	– 1.0 ± 0.4	– 0.9 ± 0.3	NS	RA SRa (1/s)	– 0.9 ± 0.3	– 0.6 ± 0.3	0.03

*HCM* hypertrophic cardiomyopathy, *EF total* total emptying fraction, *EF conduit* conduit emptying fraction, *EF booster* contractile emptying fraction, *εs* total strain, *εe* conduit strain, *εa* contractile strain, *SRs* total strain rate, *SRe* conduit strain rate, *SRa* contractile strain rate, *LAV max* indexed maximum LA volume, *LAV min* indexed minimum LA strain, *LAV pac* indexed LA volume just before atrial contraction, *RAV max* indexed maximum RA volume, *RAV min* indexed minimum RA strain, *RAV pac* indexed RA volume just before atrial contraction, *NS* not significant

Right atrial minimal volume (*p* < 0.01) and volume just before contraction (*p* = 0.01) were higher in HCM subjects than in the controls. All emptying fractions were decreased in patients with HCM compared with the controls (*p* < 0.01–0.04). Moreover, the majority of markers of atrial deformation (*p* < 0.01–0.03), with the exception of the strain rate reservoir component (*p* = NS), were reduced in children with HCM compared with the control subjects.

### LA dynamics in children with and without LVOTO

**Fig. 2:**
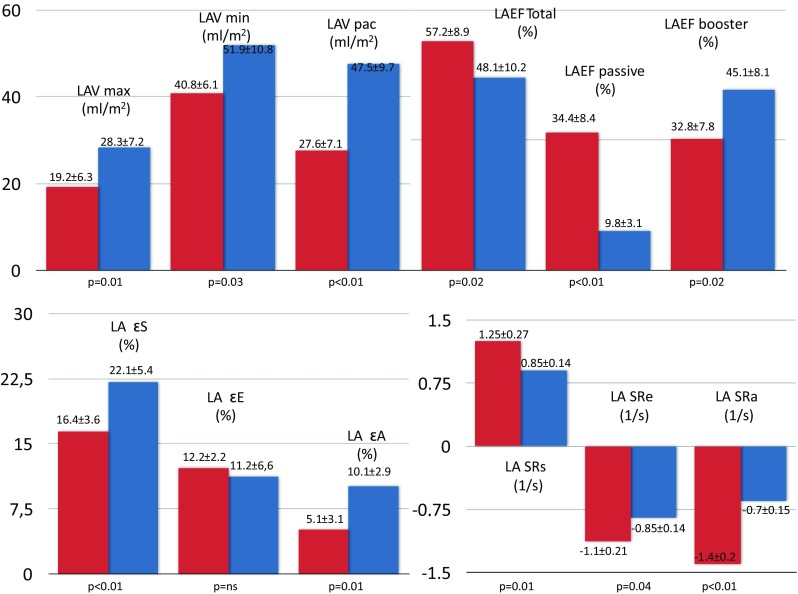
Comparisons of left atrial volumetric, emptying and mechanical components in children with and without LVTO. HCM hypertrophic cardiomyopathy, LAEF left atrial emptying fraction, ε strain, SR strain rate, LAV max maximum left atrial volume, LAV min minimum left atrial volume, LAV pac left atrial volume just before atrial contraction

All LA volumes were higher in children with LVOTO compared with children without LVOTO (*p* < 0.01–0.03). Furthermore, the reservoir (*p* = 0.02) and conduit (*p* < 0.01) emptying fraction were decreased in children with LVOTO. It is noteworthy that the active booster contractile function (*p* = 0.02) as well as the reservoir (*p* < 0.01) and booster (*p* = 0.01) strain mechanical components were higher in children with vs without LVOTO. All other emptying fractions and strain rates (*p* < 0.01–0.04) were significantly reduced in children with LVOTO (Fig. 2).

### RA dynamics in children with and without LVOTO

**Fig. 3:**
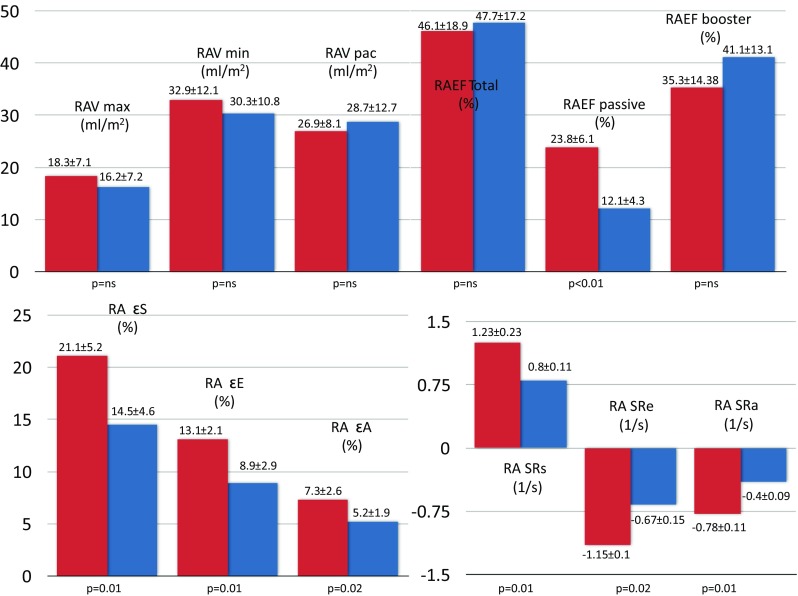
Comparisons of right atrial volumetric, emptying and mechanical components in children with and without LVTO. HCM hypertrophic cardiomyopathy, RAEF right atrial emptying fraction, ε strain, SR strain rate, RAV max maximum right atrial volume, RAV min minimum right atrial volume, RAV pac right atrial volume just before atrial contraction

None of the RA volumes were significantly affected by the presence of LVOTO (*p* = NS). Moreover, there were no differences in booster pump or total RA contractile functions between subjects with and without LVOTO (*p* = NS). On the other hand, all deformation indices of RA were significantly lower in patients with LVOTO than in subjects without LVOTO (*p* < 0.01–0.02) (Fig. 3).

### Association of biatrial function with LVOTO, ventricular mass and LV fibrosis

The associations of indices of LA and RA dynamics with the amount of fibrosis, the extent of hypertrophy and the degree of LVOTO are summarised in Tables 4 and 5, respectively. Nearly all of the LA dynamics markers attained a significant association with the LVOT gradient (*p* < 0.01–0.04). On the other hand, the LA volume prior to atrial contraction (*p* = 0.04), total volume (*p* = 0.04) and booster (*p* = 0.03) fractional volume change were related to LV mass. Lastly, only the LA conduit emptying fraction (*p* = 0.03) could be linked to the amount of LV fibrosis.

**Table 4:** Association of indices of left atrial dynamics with left ventricular outflow gradient, left and right ventricular mass indexed for body surface area and extent of fibrosis

	LVOT gradient		LGE%LV		LVMI		RVMI	
	β	*p*	β	*p*	β	*p*	β	*p*
LAV mini (ml/m^2^)	0.36	0.01	0.05	NS	0.06	NS	0.11	NS
LAV max (ml/m^2^)	0.32	0.04	0.12	NS	– 0.0	NS	0.02	NS
LAV pac (ml/m2)	0.51	< 0.01	– 0.12	NS	– 0.13	0.04	0.05	NS
LAEF total (%)	– 0.34	0.02	0.01	NS	– 0.14	0.04	0.10	NS
LAEF conduit (%)	– 0.58	< 0.01	– 0.22	0.03	0.11	NS	0.04	NS
LAEF booster (%)	– 0.34	0.02	0.11	NS	– 0.31	0.03	– 0.09	NS
LA εS (%)	– 0.42	0.03	0.12	NS	– 0.01	NS	0.01	NS
LA εE (%)	– 0.04	NS	– 0.06	NS	0.13	NS	0.04	NS
LA εA (%)	– 0.58	< 0.01	0.09	NS	0.04	NS	– 0.02	NS
LA SRs (1/s)	– 0.39	0.01	0.08	NS	0.09	NS	0.12	NS
LA SRe (1/s)	0.74	< 0.01	– 0.13	NS	– 0.08	NS	– 0.08	NS
LA SRa (1/s)	0.84	< 0.01	– 0.03	NS	0.06	NS	0.05	NS

*LVMI* left ventricular mass indexed for body surface area, *RVMI* right ventricular mass indexed for body surface area, *LA* left atrial, *LV* left ventricular, *LGE* late gadolinium enhancement, *LGE%LV* amount of fibrosis as a percentage of LV mass, *EF total* total emptying fraction, *EF conduit* conduit emptying fraction, *EF booster* contractile emptying fraction, *εs* total strain, *εe* conduit strain, *εa* contractile strain, *SRs* total strain rate, *SRe* conduit strain rate, *SRa* contractile strain rate, *LAV max* indexed maximum LA volume, *LAV min* indexed minimum LA strain, *LAV pac* indexed LA volume just before atrial contraction, *NS* not significant

**Table 5:** Association of indices of right atrial dynamics with left ventricular outflow gradient, left and right ventricular mass indexed for body surface area and extent of fibrosis

	LVOT gradient		LGE%LV		LVMI		RVMI	
	β	*p*	β	*p*	β	*p*	β	*p*
RAV mini (ml/m^2^)	0.19	NS	– 0.12	NS	0.38	0.01	0.03	NS
RAV max (ml/m^2^)	0.08	NS	– 0.21	0.03	0.06	NS	0.04	NS
RAV pac (ml/m^2^)	– 0.03	NS	– 0.16	0.03	0.02	NS	0.01	NS
RAEF total (%)	– 0.11	NS	– 0.06	NS	– 0.21	0.02	– 0.11	NS
RAEF conduit (%)	– 0.54	< 0.01	0.04	NS	– 0.03	NS	– 0.09	NS
RAEF booster (%)	– 0.12	NS	0.04	NS	– 0.31	0.01	– 0.03	NS
RA εS (%)	– 0.37	0.01	0.12	NS	– 0.05	NS	– 0.01	NS
RA εE (%)	– 0.25	0.04	– 0.14	NS	– 0.04	NS	0.03	NS
RA εA (%)	– 0.24	NS	0.02	NS	– 0.27	0.04	0.07	NS
RA SRs (1/s)	– 0.43	0.02	– 0.12	NS	– 0.15	NS	0.03	NS
RA SRe (1/s)	0.78	< 0.01	– 0.07	NS	0.11	NS	0.11	NS
RA SRa (1/s)	0.76	< 0.01	0.07	NS	0.01	NS	0.08	NS

*LVMI* left ventricular mass indexed for body surface area, *LVMI* left ventricular mass indexed for body surface area, *RVMI* right ventricular mass indexed for body surface area, *RA* right atrial, *LV* left ventricular, *LGE%LV* amount of fibrosis as a percentage of LV mass, *LGE* late gadolinium enhancement, *EF total* total emptying fraction, *EF conduit* conduit emptying fraction, *EF booster* contractile emptying fraction, *εs* total strain, *εe* conduit strain, *εa* contractile strain, *SRs* total strain rate, *SRe* conduit strain rate, *SRa* contractile strain rate, *RAV max* indexed maximum RA volume, *RAV min* indexed minimum RA strain, *RAV pac* indexed RA volume just before atrial contraction, *NS* not significant

None of the RA volumes and only the RA conduit (*p* < 0.01) fractional function exhibited an association with the degree of LVOTO (*p* = NS). However, all of the markers of RA deformation, with the exception of the strain booster component (*p* = NS), exhibited an association with LVOT gradient (*p* < 0.01–0.04). The maximum RA volume (*p* = 0.03) and volume before atrial contraction (*p* = 0.03) were associated with the extent of LV fibrosis. Finally, the minimum RA volume (*p* = 0.01) and the total (*p* = 0.01) and booster (*p* = 0.01) pump functions with strain rate booster component (*p* = 0.04) were linked to the LV mass.

### Association of biatrial function and indices of biventricular diastolic function

None of the indices of RV diastolic function had detectable association with LA dynamics and vice versa—none of transmitral velocities was connected with RA dynamics (*p* = NS). Only maximal RA volume was linked to transtricuspid A wave velocity (β = – 0.25, *p* = 0.02). On the other hand, transmitral A wave velocity was associated with maximal LA volume (β = – 0.21, *p* = 0.02) and E wave velocity with LA volume just before contraction (β = 0.37, *p* = 0.01).

### Reproducibility

The indices of reproducibility of both atrial volumes and function as well as LV fibrosis were reasonably good. For all components of biatrial function the intraobserver ICCs ranged from 0.81 to 0.98 (CoV = 0.11–0.84) and interobserver ICCs ranged 0.78 to 0.94 (CoV = 0.21–1.62). The ICCs and CoVs for the intraobserver and interobserver reproducibility regarding the quantification of LV fibrosis were 0.98 (CoV = 5.42) and 0.99 (CoV = 6.02), respectively.

## Discussion

The primary findings of our work are:

1. The majority of biatrial dynamics were severely compromised in children with HCM compared with healthy controls.
2. The degree of LVOTO was predominantly associated with the volumetric and functional indices of LA performance.
3. The RA volumes and contractile functions were affected by LV fibrosis and mass, and the RA mechanical components were related to the degree of LVOTO.

### Study population

In this study we compared atrial performance indices in HCM children versus controls. The control group was not age matched. This was mainly due to ethical concerns, which limited the recruitment of healthy children for CMR examination. For this reason, a limited number of studies recruited entirely healthy juvenile cohorts who underwent CMR [18, 19]; but only Robbers–Visser et al.’s [19] included a paediatric population with a wide age spectrum. Consequently, the vast majority of previous paediatric CMR works did not include any control groups at all [20] or included control subjects with clinically indicated CMR scans [21]. However, in patients with clinical suspicion of HCM or arrhythmogenic right ventricular cardiomyopathy and normal CMR scan, the preclinical stages of the disease cannot be excluded. Furthermore, fibrosis has been found in adult subjects with preclinical HCM in whom no LV hypertrophy was noted [22]. Therefore, the question may arise whether the observed reduction in atrial performance is more a result of younger age rather than cardiac disease. Bhatla et al. showed that LA volume indexed for BSA remained constant throughout infancy, childhood and adolescence [23]. What is more, Kutty et al. also reported a marked rate of maturational changes in biatrial strains and strain rates during the first year of life, reaching normal adult values by adolescence [24]. This data suggests that age-matched controls would have very similar values of volumetric and mechanical indices of atrial performance as our control population of healthy young adults.

### Left atrial function

In the human heart, it appears that LV systolic function largely influences LA reservoir function while LV relaxation and compliance are key modulators of atrial conduit and contractile functions [15, 24, 25]. Previous studies of echo and FT CMR in HCM adult patients consequently presented the reduction of all indices of LA dynamics [15, 26]. Our results revealed that the majority of LA volumetric and functional indices were significantly compromised in HCM children compared with healthy controls. Previous works found that conduit function was impaired in early stages of the disease [15]. Furthermore, LA reservoir volumetric function constantly exacerbates during progressive impairment of LV compliance, an increase in LV end-diastolic pressure and an advance of heart failure [15, 27]. A reduction of conduit functional components with preserved reservoir function indices in our population suggests that the severity of the disease was rather moderate. In fact, the mean NYHA class in our population was 1.7 ± 0.5.

Previous studies have identified diastolic dysfunction as a prominent factor of LA remodelling and dysfunction in adult populations [26–31]. In our study, the magnitude of LA dynamics impairment appeared to be similar to that found by Kowallick et al. [15] and Kim et al. [31]. However, our population had much smaller amounts of substrates for LV diastolic dysfunction than the populations of either Kowallick et al. or Kim et al. That finding may imply that the grade of LV compliance is not the primary factor responsible for LA functional abnormalities in a juvenile HCM population. Accordingly, we found that neither the amount of intestinal fibrosis nor hypertrophy but rather LVOTO severity was associated with both LA volume and function. Presumably, the small amounts of fibrosis and hypertrophy, as substrates for the development of diastolic dysfunction, were unable to result in increased LV loading conditions in juveniles with HCM. The haemodynamic effect of LVOTO (e.g. severe pressure overload or a decrease of coronary blood flow) appears to play a more important role in the development of LA malfunction in our population. Presumably, the progressive nature of hypertrophy and fibrosis during growth leads to the deterioration of diastolic function and escalates the effects of LVOTO and finally results in further impairment of LA function.

### Right atrium

Our contemporary understanding of atrial physiology and function stems predominantly from studies of the left adult heart. It appears that RA size and performance are likely to be subject to similar regulations as its left-sided counterpart. Even so, existing reports exploring human right atrium mechanics are very sparse. To date, there have been no CMR-derived studies focused on RA dynamics in an HCM juvenile population. Willens and colleagues, in an echocardiographic study of patients with pulmonary hypertension, demonstrated a decreased RA passive function associated with increased contribution of active atrial contraction to RV filling [32]. Similar RA functional disorders have been reported in an experimental model of chronic pulmonary arterial hypertension in dogs with banded main pulmonary arteries [33]. The researchers found an analogy between the reaction of well-studied LA and RA dynamics, concluding that the decline in compliance of the respective ventricle is the presumable mechanism of deterioration of function in both atria. Therefore, it is possible that HCM-specific ultrastructural changes are not only an attribute of hypertrophied LV myocardium but instead represent a widespread process involving RV as well, despite the lack of hypertrophy. On the other hand, elevated LV end-diastolic pressure is transmitted backwards through the RV and RA, which results in further deterioration of atrial function. Consequently, we found that the causes of RA mechanics abnormalities were likely to be multifactorial; the degree of LVOTO was mostly associated with markers of RA deformation, and RA volumetric parameters were mostly related to LV mass and fibrosis. All of these findings may imply that decreased RV compliance and a backwardly transmitted pressure burden are responsible for primary stages of the disease, which includes RA dilatation and reduced emptying performance. The severe additional load generated by LVOTO leads to additional deterioration of RA mechanics.

### Biatrial performance and ventricular diastolic function

Reports analysing diastolic function in HCM children are limited to LV only. Abnormal LV and RV relaxation, based on echocardiographic transatrioventricular valve velocities and tissue Doppler measurements, was found in the majority of adult HCM patients [34, 35]. However, in those cohorts no correlations were noted between 2D echocardiographic parameters of diastolic function and strain or strain rate parameters [36]. In our study very few atrial volumes and none of the strains were associated only with transmitral or transtricuspid velocities. These findings may confirm that diastolic dysfunction is not a major factor triggering biatrial malfunction. On the other hand, it was found that assessment of diastolic dysfunction in children using traditional Doppler techniques seems to be inadequate. Poor discriminatory performance of key echocardiographic indices and large range of normal paediatric reference values allows diagnosis of disturbed ventricular relaxation in only a small proportion of patients [37]. That may suggest that the true incidence and magnitude of diastolic dysfunction in children with HCM are not known. Further studies with novel techniques and/or modalities are needed to confirm the connection between abnormal ventricular relaxation and atrial performance in the paediatric HCM population.

### Limitations of the study

The limitations of our study are mostly inherent to its design. This study recruited a relatively small sample size with no genetic testing. No myocardial T1 mapping was available during data acquisition; thus, fibrosis was quantified using classical LGE technique and was limited to LV myocardium only. Also, data on diastolic function of healthy subjects was not available; hence, the comparison of ventricular relaxation indices between HCM children and controls was not possible.

## Conclusions

The majority of biatrial volumetric and functional indices were severely compromised in children with HCM. Unlike in adults, the magnitude of LA volumetric and mechanical malfunction in children appears to be related to the degree of LVOTO rather than to the substrates of diastolic dysfunction. On the other hand, RA volumes and contractile function were impacted by the amount of LV fibrosis and mass, while the RA mechanical components were linked to the degree of LVOTO. The indices of atrial performance may provide additional data that may serve for determining the stage of the disease. However, its clinical and prognostic usefulness in paediatric populations with HCM has yet to be determined.

## Abbreviations

BSA: Body surface area
CMR: Cardiovascular magnetic resonance
FT: Feature-tracking
HCM: Hypertrophic cardiomyopathy
ICC: Intraclass correlation coefficient
LA: Left atrium
LGE: Late gadolinium enhancement
LV: Left ventricle
LVEDV: Left ventricular end-diastolic volume
LVEF: LV ejection fraction
LVESV: LV end-systolic volume
LVM: LV mass
LVOT: Left ventricular outflow tract
LVOTO: Left ventricular outflow tract obstruction
RA: Right atrium
ROI: Region of interest

## References

[1] Maron BJ, Maron MS (2013). Hypertrophic cardiomyopathy. Lancet.

[2] Gersh BJ, Maron BJ, Bonow RO (2011). ACCF/AHA guideline for the diagnosis and treatment of hypertrophic cardiomyopathy: a report of the American College of Cardiology Foundation/American Heart Association Task Force on Practice Guidelines. Developed in collaboration with the American Association for Thoracic Surgery, American Society of Echocardiography, American Society of Nuclear Cardiology, Heart Failure Society of America, Heart Rhythm Society, Society for Cardiovascular Angiography and Interventions, and Society of Thoracic Surgeons. J Am Coll Cardiol.

[3] Maron BJ, Ferrans VJ, Henry WL (1974). Differences in distribution of myocardial abnormalities in patients with obstructive and nonobstructive asymmetric septal hypertrophy (ASH): light and electron microscopic findings. Circulation.

[4] Rakowski H, Carasso S (2007). Quantifying diastolic function in hypertrophic cardiomyopathy: the ongoing search for the holy grail. Circulation.

[5] Nistri S, Olivotto I, Betocchi S (2006). Prognostic significance of left atrial size in patients with hypertrophic cardiomyopathy (from the Italian Registry for Hypertrophic Cardiomyopathy). Am J Cardiol.

[6] Ziółkowska L, Turska-Kmieć A, Petryka J, Kawalec W (2016). Predictors of long-term outcome in children with hypertrophic cardiomyopathy. Pediatr Cardiol.

[7] Elliott PM, Anastasakis A, Borger MA (2014). ESC guidelines on diagnosis and management of hypertrophic cardiomyopathy: the task force for the diagnosis and management of hypertrophic cardiomyopathy of the European Society of Cardiology (ESC). Eur Heart J.

[8] O’Mahony C, Jichi F, Pavlou M (2014). A novel clinical risk prediction model for sudden cardiac death in hypertrophic cardiomyopathy (HC risk-SCD). Eur Heart J.

[9] Barbier P, Solomon SB, Schiller NB, Glantz SA (1999). Left atrial relaxation and left ventricular systolic function determine left atrial reservoir function. Circulation.

[10] Windram JD, Benson LN, Dragelescu A (2015). Distribution of hypertrophy and late gadolinium enhancement in children and adolescents with hypertrophic cardiomyopathy. Congenit Heart Dis.

[11] Kamal MU, Riaz IB, Janardhanan R (2016). Cardiovascular magnetic resonance imaging in hypertrophic cardiomyopathy: current state of the art. Cardiol J.

[12] Golshani S, Nasiraei-Moghaddam A (2017). Efficient radial tagging CMR exam: a coherent k-space reading and image reconstruction approach. Magn Reson Med.

[13] Khan JN, Singh A, Nazir SA, Kanagala P, Gershlick AH, McCann GP (2015). Comparison of cardiovascular magnetic resonance feature tracking and tagging for the assessment of left ventricular systolic strain in acute myocardial infarction. Eur J Radiol.

[14] Kowallick JT, Kutty S, Edelmann F (2014). Quantification of left atrial strain and strain rate using cardiovascular magnetic resonance myocardial feature tracking: a feasibility study. J Cardiovasc Magn Reson.

[15] Kowallick JT, Silva Vieira M, Kutty S (2017). Left atrial performance in the course of hypertrophic cardiomyopathy: relation to left ventricular hypertrophy and fibrosis. Invest Radiol.

[16] Hudsmith LE, Cheng AS, Tyler DJ (2007). Assessment of left atrial volumes at 1.5 Tesla and 3 Tesla using FLASH and SSFP cine imaging. J Cardiovasc Magn Reson.

[17] Lang RM, Bierig M, Devereux RB (2005). Recommendations for chamber quantification: a report from the American Society of Echocardiography's Guidelines and Standards Committee and the Chamber Quantification Writing Group, developed in conjunction with the European Association of Echocardiography, a branch of the European Society of Cardiology. J Am Soc Echocardiogr.

[18] Lorenz CH, Walker ES, Morgan VL, Klein SS, Graham TP (1999). Normal human right and left ventricular mass, systolic function, and gender differences by cine magnetic resonance imaging. J Cardiovasc Magn Reson.

[19] Robbers-Visser D, Boersma E, Helbing WA (2009). Normal biventricular function, volumes, and mass in children aged 8 to 17 years. J Magn Reson Imaging.

[20] Smith BM, Dorfman AL, Yu S (2014). Relation of strain by feature tracking and clinical outcome in children, adolescents, and young adults with hypertrophic cardiomyopathy. Am J Cardiol.

[21] Bogarapu S, Puchalski M, Everitt M (2016). Novel cardiac magnetic resonance feature tracking (CMR-FT) analysis for detection of myocardial fibrosis in pediatric hypertrophic cardiomyopathy. Pediatr Cardiol.

[22] Strijack B, Ariyarajah V, Soni R (2008). Late gadolinium enhancement cardiovascular magnetic resonance in genotyped hypertrophic cardiomyopathy with normal phenotype. J Cardiovasc Magn Reson.

[23] Bhatla P, Nielsen JC, Ko HH, Doucette J, Lytrivi ID, Srivastava S (2012). Normal values of left atrial volume in pediatric age group using a validated allometric model. Circ Cardiovasc Imaging.

[24] Kutty S, Padiyath A, Li L (2013). Functional maturation of left and right atrial systolic and diastolic performance in infants, children, and adolescents. J Am Soc Echocardiogr.

[25] Stefanadis C, Dernellis J, Toutouzas P (2001). A clinical appraisal of left atrial function. Eur Heart J.

[26] Paraskevaidis IA, Panou F, Papadopoulos C (2009). Evaluation of left atrial longitudinal function in patients with hypertrophic cardiomyopathy: a tissue Doppler imaging and two-dimensional strain study. Heart.

[27] Prinz C, Van Buuren F, Bogunovic N, Bitter T, Faber L, Horstkotte D (2012). In patients with hypertrophic cardiomyopathy myocardial fibrosis is associated with both left ventricular and left atrial dysfunction. Acta Cardiol.

[28] Mulder BJ, van der Wall EE (2008). Size and function of the atria. Int J Cardiovasc Imaging.

[29] Yang WI, Shim CY, Kim YJ (2009). Left atrial volume index: a predictor of adverse outcome in patients with hypertrophic cardiomyopathy. J Am Soc Echocardiogr.

[30] Eshoo S, Semsarian C, Ross DL, Thomas L (2010). Left atrial phasic volumes are modulated by the type rather than the extent of left ventricular hypertrophy. J Am Soc Echocardiogr.

[31] Kim KJ, Choi HM, Yoon YE (2016). Left atrial mechanical function and global strain in hypertrophic cardiomyopathy. PLoS One.

[32] Willens HJ, Fertel DP, Qin J, Labrador AE, Lowery MH (2008). Effects of age and pulmonary arterial hypertension on the different phases of right atrial function. Int J Cardiovasc Imaging.

[33] Gaynor SL, Maniar HS, Bloch JB (2005). Right atrial and ventricular adaptation to chronic right ventricular pressure overload. Circulation.

[34] Maron BJ, Spirito P, Green KJ, Wesley YE, Bonow R, Arce J (1987). Noninvasive assessment of left ventricular diastolic function by pulsed Doppler echocardiography in patients with hypertrophic cardiomyopathy. J Am Coll Cardiol.

[35] Efthimiadis GK, Parharidis GE, Karvounis HI, Gemitzis KD, Styliadis IH, Louridas GE (2002). Doppler echocardiographic evaluation of right ventricular diastolic function in hypertrophic cardiomyopathy. Eur J Echocardiography.

[36] Williams LK, Chan RH, Carasso S (2015). Effect of left ventricular outflow tract obstruction on left atrial mechanics in hypertrophic cardiomyopathy. Biomed Res Int.

[37] Dragulescu A, Mertens L, Friedberg MK (2013). Interpretation of left ventricular diastolic dysfunction in children with cardiomyopathy by echocardiography: problems and limitations. Circ Cardiovasc Imaging.

